# Notes and News

**Published:** 1887-09-24

**Authors:** 


### NOTES AND NEWS.

COLLECTED AND COMMUNICATED.

THE sum of nearly £100 has been raised by Sunday and Saturday collections and Trade Societies' demonstrations at Christchurch, Hants, on behalf of the local hospitals.

THE local treasurer of the Edinburgh Royal Infirmary has addressed a circular letter to every minister of religion in the district of Caithness, asking for a special collection on behalf of the institution.

MR. R. A. MURRAY, Mr. John Keay, and Mr. Edward Gordon have been elected honorary surgeons to the Stockport Infirmary.

THE committee of the Victoria Park Flower Show, Portsmouth, have voted a donation of £5 to the fund of the Portsmouth aud South Hants Eye and Ear Infirmary. This is a good example which might be followed by many flower-show committees.

PHOTOGRAPHIC views give pleasure to many people, and would be highly appreciated by hospital patients. Mr. Alfred Constantine, writing to the *Birmingham Post*, makes an appeal for albums, in order that he may obtain from various sources a sufficient number of good views to fill them. When they are full he proposes to present one or more to each of the Birmingham hospitals. Enlarging upon this suggestion, we should be glad if any friends of the London and provincial hospitals who have leisure would take this matter in hand. An album for each hospital ward, suitably arranged for men, women, and children, would be a constant source of delight, besides possessing great educational value. We heartily commend the subject to those who have means and time at disposal.

THE first meeting of the fifth session of The Hospitals Association will be held in the Library of the Society of Arts, John Street, Adelphi (Charing Cross), on Wednesday, October 12th, at 8 p.m., Sir Andrew Clark, Bart., M.D. (President), in the chair, when a paper explanatory of the scheme for establishing a "National Pension Fund for Hospital Officials and Trained Nurses" will be read. All interested in this subject, and in the work of The Hospitals Association generally, are cordially invited to send their names and addresses to Mr. Howard J. Collins (the Secretary), Norfolk House, Norfolk Street, W.C., who will forward invitation cards. A discussion will follow.

ON the eve of the commencement of the fifth session of The Hospitals Association, its members are invited individually to use their influence with their friends to induce them to become members also.

THE Hospitals Association has done much in the past, and will undoubtedly do more in the future for the assistance and benefit of all concerned in the management, as well as of the nurses and patients, of hospitals and other medical charities throughout the United Kingdom.

AN increased number of members will allow of a wider field of work. Members joining now only pay 10s. 6d. subscription for remainder of this year, and receive weekly, post free, a copy of THE HOSPITAL—the journal of the Association.

THE secretary (Mr. Howard J. Collins) will be happy to give information as to the objects and work of the Association, on application either personally or by letter at Norfolk House, Norfolk Street, Strand, W.C.

A STATE procession, headed by the Mayor, Mr. Alderman Peace, J.P., and the borough justices, took place at Bridgewater on the occasion of the preaching of the annual sermon at St. Mary's in aid of the infirmary. The morning preacher was the Rev. E. Tapsfield, M.A., vicar of Nether-Stowey. It is somewhat unusual for the Mayor and Corporation to make a demonstration of so public a character in aid of the local hospital. But such a display of interest on the part of the *patres conscripti* of the town cannot but excite a great deal of public interest, and prove highly advantageous to the interests of the institution. Other towns might very well follow the example of Bridgewater.

A GREATLY increased amount of work, with hardly any additional expenditure, is the principal feature of the report presented to the half-yearly Board of Governors of the Bristol General Hospital. The meeting, which took place recently, was presided over by Mr. Proctor Baker, and the secretary, Mr. W. Thwaites, read the report. There is so much pressure upon the resources of the hospital that cases have to be sent away which ought to be received, but the building is in course of enlargement, and the funds are in a prosperous condition. It is hoped that the enlargement which is now in progress may be sufficient to meet the wants of the district for some considerable time.

THE following vacancies are announced this week, the amount of the salary being placed in each case after the name of the institution :—

**Resident Medical Officers.**

Birmingham General Hospital. Assistant House Surgeon.
British Hospital, Buenos Ayres. Medical Officer.—£200.
East London Hospital for Children. Two Clinical Assistants.
Female Lock Hospital, Harrow Road. House Surgeon.—£100.

**Non-resident Medical Officers.**

Bristol Royal Infirmary. Obstetric Physician.
Great Northern Central Hospital Obstetric Physician.
Sheffield General Infirmary. Two Honorary Assistant Physicians and two Surgeons.

**Matrons.**

South-Western Hospital, Stockwell.—£100, with board, etc.
Newport Infirmary and Dispensary.—£50.

**Trained Nurses.**

Darlington Fever Hospital.—£25.
South-Eastern Hospital, New Cross.—Trained Nurses, £30 to £36.
Stroud Hospital. Trained, for Male Wards.—£23.
St. Luke's Institution, Oppidans Road, N.W. Apply to Lady Principal.

---

MR. ELLIS LEVER has great faith in the virtues of sea-water. By way of stamping out the present epidemic of scarlet fever, he proposes to lay pipes from the sea-shore to London, and to give every Board-school child a sea-water bath daily. Perhaps the ratepayers might have something to say to this suggestion.

---

FOR the sum of five shillings and costs, it appears that a man is able at Oldham to contract himself out of the Vaccination Acts. At a meeting recently held in that town, Dr. Niven, the Medical Officer of Health, said he was told that vaccination in Oldham was practically a dead letter, because the fine imposed by the magistrates in case of refusal to vaccinate was a nominal one. It seems that at the present time but a small proportion of the children of Oldham are vaccinated. If other towns should follow the example of Leicester and Oldham, we may expect a good deal of light to be thrown on the question of the value of vaccination as a prophylactic.

---

OPEN spaces in London are not likely ever to be either too numerous or too extensive. We are always glad to see efforts made to retain any that may be threatened or to acquire new ones. A meeting was held at the Pembroke Schools, Kennington Green, for the purpose of protesting against the proposed purchase, out of the rates, of the Lawn, South Lambeth, and Raleigh House, Brixton, at an outlay of about £100,000. Mr. John Bennett, a member of the Lambeth vestry, presided, and the meeting was of a stormy character. A resolution was moved affirming that the purchase proposed was unnecessary and unjust, and this resolution was ultimately declared to be carried. The people of Lambeth and the neighbourhood are making a very serious mistake. The sum in consideration, when spread over the rates, amounts to an exceedingly small item for each ratepayer, whilst the value of the open space, not only to the ratepayers but to their families, is incalculable. It may be hoped that a representative meeting of the more intelligent among the inhabitants will take a wiser view of the question.

THE working men in the district of West Ham are making energetic efforts to establish a hospital for themselves ; and in aid of the funds a fête and cricket match took place recently between the local tradesmen and the police of the K Division.

---

BELFAST considers £226 10s. 6d. to be an adequate sum to raise on its Hospital Saturday. Belfast has a population of about 200,000. The people, therefore, gave a little more than a farthing each towards the support of their hospital on the occasion of their second great annual effort. Belfast is clearly a generous town.

---

THE people of Nottingham are arguing out the question of a new infectious hospital with some warmth. It is everybody's experience that a well-manned and efficient fever-hospital is not only of the greatest possible advantage to poor persons who may be attacked with fever, but is perhaps the best protector of the public health against the spread of infectious diseases which has ever been devised.

---

JAMES HUTTON, a young cabman, residing at Paddington, was seriously injured on Hospital Saturday whilst rendering voluntary services to the Fund, by driving round to the various stalls with tables and collecting-boxes. He was taken to St. Mary's Hospital, and has since recovered, though he is not yet fit for work. His brother cabmen have bestirred themselves on his behalf, and a concert was given on Wednesday at the Grafton Hall, Grafton Street, under the management of Mr. Joseph Hoffman, the proceeds of which will be devoted to his use.

---

A SMALL attendance at a half-yearly hospital meeting indicates a satisfied public, according to Colonel Thorneycroft. That is probably the view Mark Tapley would have taken of the matter. The gallant Colonel presided at the half-yearly meeting of the Wolverhampton and Staffordshire Hospital on the 13th inst., and the absence of special features in the report, as well as the paucity of governors present, were interpreted by him as quite happy events. The Colonel may be congratulated on his easy philosophy.

---

A CASE recently occurred at Brighton which should act as a warning to hospital dispensers and chemists. Mrs. Haines, a boarding-house keeper, brought an action against Messrs. Savory and Moore for having wrongly labelled a lotion, which the lady said had caused injury to her eyes. Two prescriptions, one a lotion to be applied behind the ears, and the other a lotion for the eyes, were handed to Messrs. Savory and Moore. The prescriptions were properly made up, but the "finisher" interchanged the labels. Mrs. Haines applied the lotion intended for the ears to her eyes, and it caused great pain and inflammation. Mr. Critchett, the oculist, who was called for the defence, stated that no permanent or other injury was likely to result. Notwithstanding this, Mrs. Haines was awarded damages to the amount of £62.

Mr. Horace Jefferies has resigned the office of house-surgeon at the Bridgnorth Infirmary, after an occupancy of three years. The committee passed a resolution on the occasion, which is highly honourable alike to themselves and to Mr. Jefferies. In it they state that they accept Mr. Jefferies' resignation with great regret; that he has performed his duties with "industry, care, zeal, accuracy, firmness, and kindness"; that at the time of his election, three years ago, the infirmary was in a very unsatisfactory state, that it was mainly through his representations that they were made aware of the defects of the institution, and by his loyal and patient co-operation that they were able to reform them.

A head-master gives his experience thus in the *Pall Mall Gazette*:—"In a general knowledge paper set for one of the middle forms of this school the boys were asked to state what they knew of a list of persons of the present day; one of the names given was that of John Ruskin. It may interest you to see three of the answers. Question: What do you know of the following names? John Ruskin, etc., etc.—Answer 1: John Ruskin was lately Prime Minister. Answer 2: Mr. Ruskin is the editor of *Punch*. Answer 3: Mr. John Ruskin is a gentleman who writes to *Punch*, and uses very bad language. Another answer was that Sir Gilbert Scott was father of Sir Walter."

The annual consumption of lager beer in New York city is set down at about 6,000,000 barrels, which, to say nothing about the other forms of intoxicating liquors consumed, gives about five barrels for every man, woman, and child in the city. The first cost of the intoxicating liquors sold in the United States is annually about £170,000,000, which is more than the cost of bread and meat combined, more than that of the woollen and cotton goods, boots and shoes, and sugar and molasses combined, more than half that of all the steel and iron and sawed lumber combined, ten times as much as the cost of all the public education of the land, and one hundred and fifty times as much as the cost of all our home and foreign missions. The drink bill of the United States is more than £400,000 a day for every day in the year.

Lancashire is not to be outdone by the neighbouring county of Yorkshire in enthusiasm on behalf of its hospitals. In aid of the funds of the Blackburn Infirmary a grand *fête* was given on the 17th inst. in the Alexandra Meadows, Blackburn. The *fête* consisted of an entertainment in drill, dumb-bells, Indian clubs, and wand exercises by the school children of Blackburn, under the direction of Sergeant-Major Galbally, R.A. A gymnastic display was given by members of the Blackburn troop of the Duke of Lancaster's Own Yeomanry Cavalry. The performance consisted of cavalry sword exercises, fencing, parallel bars, boxing, sword exercises, Indian clubs, horizontal bar, single-stick, and "Ye goode olde Englishe sporte of quarter staffe." The display was under the captaincy of Mr. Wheatcroft.

The Metropolitan Asylums Board has changed its mind, and resolved to open the hospital at Winchmore Hill for the reception of scarlet fever convalescents. It may be hoped that there will be no needless delay in furnishing and getting the wards ready for use. The Board has not acted with so much wisdom and promptitude as might have been wished, but it has shown a commendable desire to save the public money. The constitution of this particular body is not such as to warrant a belief in its capacity for dealing with grave emergencies with judgment and despatch. That, however, is not the fault of the Board, but of its creators. Newspaper critics are, no doubt, very clever persons, and public bodies are, of course, fair game for a little display of wit and superior ability. But it may be doubted whether the writers of leaders and paragraphs who have made a target of the Board for the last few weeks would have acted with any more wisdom had they themselves been called upon to deal with the question. The Board have now resolved to take the right course, and it is permissible to urge upon them the necessity for moving with all possible speed.

The epidemic of fever which is now upon us is becoming seriously alarming. On Tuesday fifty new cases were admitted into the hospitals of the Metropolitan Asylums Board, and on Monday forty-nine. All the London hospitals, except Stockwell, are now filled to their utmost capacity. 1,400 patients were under treatment on Wednesday at those hospitals, of which 1,221 were scarlet-fever cases. The Winchmore Hill Convalescent Hospital is not yet ready for occupation; but as operations have been commenced it may be presumed they will be pushed forward with all possible speed. In spite of the alarming epidemic, the London death-rate for last week was lower than for any week since the commencement of the year, being only 15.7.

Miss Noble, governess to Dr. Turtle, of Woodford, was killed on Sunday evening by some young men driving a horse and trap furiously along the road. Whilst the people were leaving the parish church these young fellows urged their horse at full speed through the crowd. Miss Noble and Miss Ellis, who were unable to get out of the way, were both knocked down, the former being run over, with results which proved fatal in half an hour. The young men drove rapidly off, and cannot be discovered. Every person should feel it a duty to aid the authorities in their search for these reckless miscreants. Many London suburbs are in constant terror all the Sunday long in consequence of men of this class, who have neither sense nor feeling. If the scoundrels are caught the driver should either be hanged or sent to penal servitude for life as a warning to others.

A baby, four months old, was worried to death by a ferret at Misterton, near Yeovil, on Monday night. The ferret, which had apparently escaped from its cage, found its way into the bed where the child was lying asleep. When discovered, the infant had one of its eyes torn out, and its face was lacerated in a terrible manner. The ferret belonged to a labourer, named Osborne. Labourers, when they are not rat-catchers, can have no legitimate use for ferrets, and if a heavy tax were put upon them, the number of these vicious and untamable brutes would soon be diminished.

